# Behavioral and Psychological Symptoms Impact Clinical Competence in Alzheimer’s Disease

**DOI:** 10.3389/fnagi.2017.00182

**Published:** 2017-06-16

**Authors:** Elodie Bertrand, Eelco van Duinkerken, J. Landeira-Fernandez, Marcia C. N. Dourado, Raquel L. Santos, Jerson Laks, Daniel C. Mograbi

**Affiliations:** ^1^Department of Psychology, Pontifícia Universidade Católica – Rio (PUC-Rio)Rio de Janeiro, Brazil; ^2^Department of Medical Psychology, VU University Medical CenterAmsterdam, Netherlands; ^3^Diabetes Center/Department of Internal Medicine, VU University Medical CenterAmsterdam, Netherlands; ^4^Institute of Psychiatry—Center for Alzheimer’s Disease, Federal University of Rio de Janeiro (UFRJ)Rio de Janeiro, Brazil; ^5^Post Graduation Program on Translational Biomedicine, Universidade do Grande Rio (Unigranrio)Caxias, Brazil; ^6^Institute of Psychiatry, Psychology & Neuroscience, King’s College LondonLondon, United Kingdom

**Keywords:** BPSD, clinical capacity, decision making, consent to treatment, dementia, Alzheimer’s disease

## Abstract

Decision-making is considered a fundamental aspect of personal autonomy and can be affected in psychiatric and neurologic diseases. It has been shown that cognitive deficits in dementia impact negatively on decision-making. Moreover, studies highlighted impaired clinical competence in neuropsychiatric disorders, such as schizophrenia and bipolar disorder. In this context, the current study explored the relationship between behavioral and psychological symptoms of dementia (BPSD) and clinical competence, especially the capacity to consent to treatment, in Alzheimer’s disease (AD). Seventy-one patients with mild to moderate AD participated, completing assessments for capacity to consent to treatment, general cognition and neuropsychiatric disturbances. For each neuropsychiatric symptom, patients with and without the particular disturbance were compared on the different subscales of the MacArthur Competence Tool for Treatment (MacCAT-T; Understanding, Appreciation, Reasoning and Expression). The results showed that patients presenting delusions, as well as apathetic patients, had a lower ability to express a clear treatment choice compared to patients without these symptoms. By contrast, patients with dysphoria/depression had higher scores on this variable. Additionally, AD patients with euphoria had more difficulties discussing consequences of treatment alternatives compared to patients without this disturbance. None of the differences were confounded by global cognition. There were no between-group differences in clinical decision-making for patients with hallucinations, agitation/aggression, anxiety, irritability, disinhibition and aberrant motor behavior. These findings highlight the importance of taking BPSD into account when assessing decision-making capacity, especially clinical competence, in AD. Furthermore, reducing BPSD may lead to better clinical competence in patients with AD, as well as to improvements in patients and caregivers’ quality of life.

## Introduction

In the context of contemporary clinical practice, in which the patients’ active participation in medical decisions is valued, the question of decision-making capacity, especially the competence to consent to treatment has become central. Moreover, treatment consent capacity, which refers to the ability to accept an offered treatment, refuse it, or select among alternatives, has important clinical, legal and ethical implications (President’s Commission for the Study of Ethical Problems in Medicine and Biomedical and Behavioral Research, 1982; Tepper and Elwork, 1984). Indeed, it is considered a fundamental aspect of personal autonomy and its careful assessment is essential to find the balance between autonomy for patients who are able to make their own decisions and protection for those with diminished decisional capacity (Berg et al., 2001; Berghmans and Widdershoven, 2003). In the case of Alzheimer’s disease (AD), these concerns seem even more relevant, since this is a condition affecting cognitive abilities critical to healthcare decision-making capacity (Marson, 2001). It has been suggested that offering the possibility to people with dementia (PwD) to participate in healthcare-related decisions may improve their well-being, quality of life and dignity compared to those excluded from taking part in decisions, who tend to be more depressed, frustrated and debilitated (Smebye et al., 2012).

Previous research exploring medical decision-making, also termed clinical competence, has shown that, compared with healthy control individuals, the treatment consent capacity of individuals with AD is reduced (Karlawish et al., 2005; Lui et al., 2012). These findings have been supported by longitudinal studies, which highlighted that clinical competence tends to decrease over time in mild cognitive impairment and AD (Moye et al., 2006; Okonkwo et al., 2008). The decrease of decisional capacity in AD has been related to cognitive decline in this population, with research focusing on cognitive predictors showing that problems with language, memory and executive function impact negatively on decisional capacity (Moye et al., 2006; Stormoen et al., 2014).

In addition to the characteristic cognitive deficits, behavioral and psychological symptoms of dementia (BPSD) are an integral part of AD (Cerejeira et al., 2012; Zhao et al., 2016). BPSD are widespread non-cognitive symptoms, including apathy, aggression, delusions, psychosis, hallucinations, anxiety, irritability, depression and sleep disorders (van der Linde et al., 2014). The presence of BPSD has important negative consequences on AD patients and their caregivers. For instance, BPSD result in premature institutionalization, increased cost of care and diminished quality of life for both patients and caregivers (Black and Almeida, 2004; Scarmeas et al., 2007; Schaller et al., 2015; Feast et al., 2016). Mitoku and Shimanouchi (2014) explored the impact of BPSD on decision making capacity in older adults and highlighted that older adults with dementia presenting BPSD have decreased decisional capacity compared to patients without BPSD. Mograbi et al. (2015) showed in a large community-based study that BPSD correlated with unawareness of memory deficits in dementia, which may negatively affect clinical capacity.

Moreover, it has been shown that decisional capacity is impaired in various neuropsychiatric disorders (for review see Rahman et al., 2001; Candia and Barba, 2011). Looking especially at clinical competence, previous research highlighted that patients with schizophrenia, bipolar mood disorder and major depression have significant impairments in decisional abilities (Grisso and Appelbaum, 1995; Howe et al., 2005; Appelbaum and Redlich, 2006). Additionally, studies demonstrated that for patients with acute psychosis, treatment consent capacity was related to the extent and severity of symptoms, especially positive symptoms such as delusions and hallucinations (Howe et al., 2005; Rutledge et al., 2008). However, for patients with chronic psychosis, Palmer et al. (2004) showed a relationship between cognitive and negative symptoms (unusual thought content, conceptual disorganization) and impaired medical decision-making capacity.

Considering the scarcity of studies in this field, the aim of the present study was to explore the relationship between behavioral disturbance and decision-making in AD by comparing patients with or without BPSD on their capacity to consent to treatment.

## Materials and Methods

### Participants

A consecutive series of 71 patients and caregivers dyads were recruited from an AD outpatient unit. We included participants diagnosed with possible or probable AD according to the Diagnostic and Statistical Manual of Mental Disorders, fourth edition (DSM-IV-TR). The clinical diagnosis of AD was made by a psychiatrist, based on clinical interviews with the patients and their caregivers, cognitive screening tests, laboratory tests and imaging studies. Inclusion criteria were: mild Clinical Dementia Rating (CDR = 1; *n* = 50) and moderate (CDR = 2; *n* = 21) dementia according to the CDR and Mini-Mental State Examination (MMSE) scores of 11–26. We excluded people with head trauma, aphasia, history of alcoholism, psychotic symptoms, epilepsy and uncontrolled medical problems such as hypertension, depression and diabetes.

The primary caregiver was defined as the main person responsible for the care of the patient. All of the caregivers had been previously informed of the AD diagnosis by the psychiatrist. The patients completed assessments about cognition and competence to consent to treatment. The caregivers provided information about the patients’ demographics, the ability to perform activities daily living (ADL), neuropsychiatric symptoms, functionality, dementia severity and had depression and burden of care assessments.

### Instruments

#### Competence to Consent to Treatment

The MacArthur Competence Tool for Treatment (MacCAT-T; adapted to Brazilian Portuguese; Santos et al., 2017) was used to assess competence to consent to treatment (Grisso et al., 1997). This scale permits to explore four different abilities: understanding, appreciation, reasoning and expression. The *Understanding* section assesses the capacity to paraphrase what has been disclosed. The *Appreciation* section assesses whether the individual acknowledges that the disclosed information applies to him or her and whether he or she recognizes the treatment possible benefits. *Reasoning* explores whether the person mentions any consequence of the treatment alternatives, comparison among alternatives and any consequences that were not mentioned in the disclosure. Finally, in the *Expression* section, the individual is supposed to offer a clear expression of a treatment choice and to explain how this choice was made. Patient responses were rated using the following scoring: 2 points for adequate; 1 point for partially sufficient; and 0 points for insufficient. Total scores in the MacCAT-T subscales ranged as follows: Understanding, 0–6; Appreciation, 0–4; Reasoning, 0–8; Expression, 0–2.

#### Cognition

For a general measure of cognitive level, the MMSE was used (Folstein et al., 1975; Bertolucci et al., 1994). The total score ranges from 0 to 30, with lower scores indicating impaired cognition. The Alzheimer Disease Assessment Scale—Cognitive Subscale (ADAS-Cog; Schultz et al., 2001), which assesses the intensity of cognitive changes, was also applied.

The ADAS-cog is 11-item scale used to assess the severity of selected areas of cognitive impairment (memory, language, orientation, reason and praxis). The maximum score is 70 and higher scores indicate poorer performance. Finally, attention and working memory were assessed with the Wechsler Digit Span Test, Forward and Backward (Wechsler, 1997; Nascimento, 2000). Scores of Digit Span Forward vary from 0 to 16, and scores of Digit Span Backward vary from 0 to 14, with higher scores indicating better performance.

#### Neuropsychiatric Symptoms

The Neuropsychiatric Inventory (NPI) was used to assess 10 neuropsychiatric disturbances commonly observed in dementia (Cummings et al., 1994; Camozzato et al., 2008). The scale evaluates the presence of delusions, hallucinations, dysphoria, anxiety, agitation/aggression, euphoria, disinhibition, irritability/lability, apathy, aberrant motor activity, night-time behavior disturbances and appetite and eating abnormalities. The NPI is administrated to the patient’s caregiver, who rates each item in relation to their frequency (1 [absent] to 4 [frequent]) and to their severity (1 [mild] to 3 [severe]). The total score can range from 0 to 144 points, with higher scores indicating more severe psychopathology. For the purpose of this study, the subscales for each symptom were used, with patients being dichotomized according to the presence of the symptom (see “Statistical Analysis” Section below).

#### Dementia Severity

To determine dementia severity, the full protocol of the CDR was used (Morris, 1993; Maia et al., 2006), with severity ranging from 0 (no dementia) to 3 (severe dementia). Only patients with CDR 1 and 2 were included in the study.

### Ethical Issues

This study was carried out in accordance with the recommendations of the Federal University of Rio de Janeiro (UFRJ)/Institute of Psychiatry Ethics Committee with written informed consent from all subjects. All subjects gave written informed consent in accordance with the Declaration of Helsinki. The protocol was approved by the Federal University of Rio de Janeiro (UFRJ)/Institute of Psychiatry Ethics Committee (Research Ethics Committee number 536.634).

### Statistical Analysis

Demographic and clinical data are presented as means with standard deviation and range for the whole group. Dichotomous variables are presented as absolute numbers with percentages.

First, exploratory Pearsons correlations investigated the relationship between cognitive variables and clinical competence. Second, patients were dichotomized into two groups per subscale of the NPI. The first group included patients that did not report any symptoms on that particular subscale, whereas the second group comprised all the participants that had a score of 1 or more on that specific NPI subscale. Then, for each of the 10 NPI subscales, using Student *t*-tests for independent samples, we compared the mean scores on each scale of the MacCAT-T between the two groups. In case of a significant difference between the groups, the test was repeated now correcting for the ADAS-Cog score, to rule out the effect of global cognition differences. Additionally, to ensure that the significant differences between groups were not due to differences in sex, level of education, age and dementia severity, we tested differences according to these variables for each MacCAT-T section. For this purpose, independent samples *t*-tests were used, with patients being dichotomized according to sex, CDR (1: mild vs. 2: moderate dementia), educational level (1–9 years vs. 10+) and age (61–74 years old vs. 75+).

All tests were performed in SPSS20 (IBM-SPSS, Chicago, IL, USA). A *p* < 0.05 was considered statistically significant.

## Results

### Participant Characteristics

A total of 71 patients with AD participated in this study. Sample characteristics and scores in the MacCAT-T can be found in Table 1. MacCAT-T results are consistent with previous data from patients with dementia, showing lower scores for Understanding and Reasoning when compared to a group of healthy controls (Moye et al., 2004).

**Table 1:** Participant characteristics and scores on the neuropsychiatric inventory (NPI) and MacArthur Competence Tool for Treatment (MacCAT-T).

	Alzheimer’s disease patients (*n* = 71)
	Mean ± SD (Range)
*Demographic variables*	
Age (years)	78.2 ± 6.3 (61–93)
Sex*	42/29 (59.2/40.8)
Education (years)	7.5 ± 3.9 (1–15)
MMSE	19.5 ± 4.0 (11–27)
ADAS Cog	24.5 ± 8.9 (8–48)
Digits—Forward	7.6 ± 2.6 (4–15)
Digits—Backward	3.2 ± 1.7 (0–9)
CDR**	50/21 (70.4/29.6)
Disease duration (years)	5.3 ± 3.5 (1–16)
Disease onset age (years)	72.8 ± 7.3 (54–88)
*MacCAT-T scales*	
Understanding	4.3 ± 0.9 (2.15–6)
Appreciation	3.2 ± 1.0 (0–4)
Reasoning	3.3 ± 1.5 (0–6)
Expression	1.8 ± 0.6 (0–2)

*n female/male, %; **n CDR 1/CDR 2, %.

The frequency of patients with neuropsychiatric symptoms can be seen in Table 2. In addition, no significant differences in the MacCAT-T subscales were found for sex, level of education, age and disease severity.

**Table 2:** Frequency of NPI symptoms.

	AD patients (*n* = 71) *n* without/with symptom (%)
*NPI symptoms*	
Hallucinations	61/10 (85.9/14.1)
Delusions	58/13 (81.7/18.3)
Agitation/aggression	51/20 (71.8/28.2)
Dysphoria/depression	33/38 (46.5/53.5)
Anxiety	36/35 (50.7/49.3)
Irritability	39/32 (54.9/45.1)
Disinhibition	56/15 (78.9/21.1)
Euphoria	67/4 (94.4/5.6)
Apathy	36/35 (50.7/49.3)
Aberrant motor behavior	52/19 (73.2/26.8)

### Correlation Analyses

Correlations between cognitive variables and clinical competence can be seen in Table 3. *Understanding* was significantly correlated MMSE (*r* = 0.28, *p* < 0.05), ADAS-Cog (*r* = −0.30, *p* < 0.05) and Digit-backward (*r* = 0.24, *p* < 0.05). There was a significant correlation between *Reasoning* and ADAS-Cog (*r* = −0.28, *p* < 0.05), as well as between *Reasoning* and Digit-backward (*r* = 0.25, *p* < 0.05). There were no significant associations between *Appreciation* score and the cognitive variables, neither between *Expression* and these variables.

**Table 3:** Correlations between cognitive variables and clinical competence.

Variable	Understanding	Appreciation	Reasoning	Expression
MMSE	**0.28**	0.07	0.21	0.07
ADAS-Cog	**−0.30**	−0.09	**−0.28**	−0.12
Digits—Forward	0.21	−0.02	0.12	−0.11
Digits—Backward	**0.24**	0.14	**0.25**	−0.06

*Significant results are presented in bold*.

### NPI Subscales

**Figure 1:**
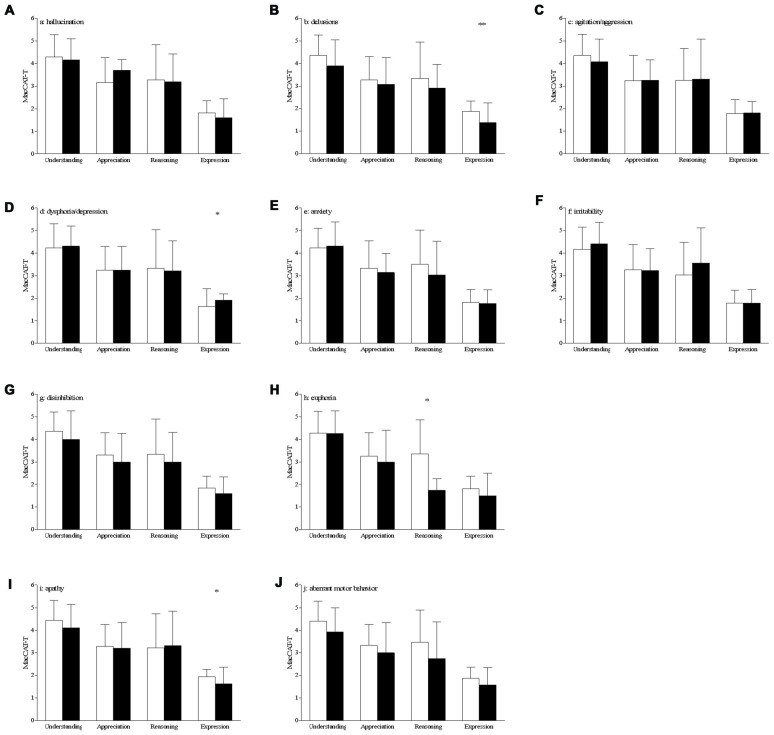
Differences on MacArthur Competence Tool for Treatment (MacCAT-T) subscales according to the presence of behavioral and psychological symptoms of dementia (BPSD; **A–J**) bar graph of the mean with standard deviation of the MacCAT-T subscales per subscale of the neuropsychiatric inventory (NPI). White bars represent the patients with a score of 0 on that domain, black bars those patients with a score of 1 or higher on that domain; *p < 0.05, **p < 0.01; significant differences can be seen for delusions, dysphoria and apathy (expression of choice) and for euphoria (reasoning).

Patients with delusions had a lower score on the *expression* scale of the MacCAT-T (without: 1.9 ± 0.5 vs. with: 1.4 ± 0.9, *t*_(69)_ = 2.91, *p* = 0.005, Figure 1B). This remained statistically significant after correction for the ADAS-Cog score (*p* = 0.006). *Expression* was also affected in patients with dysphoria/depression, who showed higher scores (without: 1.6 ± 0.8 vs. with: 1.9 ± 0.3, *t*_(69)_ = −2.10, *p* = 0.039, Figure 1D), which remained significant after correction for the ADAS-Cog score (*p* = 0.038). This variable was also affected in apathetic patients, who had a lower score on *expression* as compared with the patients scoring 0 on this NPI subscale (without 1.9 ± 0.3 vs. with: 1.6 ± 0.7, *t*_(69)_ = 2.35, *p* = 0.021, Figure 1I). After correcting for the ADAS-Cog score, this difference remained statistically significant (*p* = 0.023).

AD patients with euphoria had a lower score on the *reasoning* scale of the MacCAT-T (without: 3.4 ± 1.5 vs. with: 1.7 ± 0.5, *t*_(69)_ = 2.12, *p* = 0.038, Figure 1H). However, this effect is not statistically significant after correction for the ADAS-Cog scores (*p* = 0.082).

There were no between-group differences in MacCAT-T scores for patients with hallucinations (Figure 1A), agitation/aggression (Figure 1C), anxiety (Figure 1E), irritability (Figure 1F), disinhibition (Figure 1G) and aberrant motor behavior (Figure 1J; in all cases, *p* > 0.05).

## Discussion

The aim of this exploratory study was to investigate the relationship between behavioral symptoms and capacity of clinical decision-making in AD. The results indicate that patients with symptoms of delusions and apathy exhibit impaired *expression* of choice in comparison to patients without these symptoms. By contrast, patients with symptoms of dysphoria and depression have higher scores on this particular subscale relative to patients without symptoms. None of the differences were confounded by global cognition as measured by the ADAS-Cog. Additionally, patients with euphoria score lower on the subscale of *reasoning* compared to patients without these symptoms, however, when correcting for global cognition using the ADAS-Cog, this result is no longer significant.

Worse performances on the *expression* section of the MacCAT-T were seen for patients with both delusion and apathy. The result for delusions may be explained by an error in reality monitoring, which may impair the ability of patients to clearly express and explain treatment choices. For apathy, increasing space has been given to the role of emotional processes in decision making (Kahneman and Tversky, 1979; Damasio, 1994). It is possible that apathetic patients cannot rely on affective information to express their treatment choices. Apathy is also characterized by lack of motivated behavior, including the difficulty to engage in a cognitively demanding task (Marin, 1990) and this may be a potential reason for the lower scores of apathetic patients when asked to express a treatment choice. Indeed, the *expression* section of the MacCAT-T appears as a highly demanding task in term of motivational resources. Additionally, the impaired capacity to clearly express a treatment choice may affect decision making. For example, patients impaired in their ability to express their decisions may be excluded from the decision-making process by caregivers, contributing to malignant social psychology, which is defined by caregivers’ behaviors that undermine the personhood and wellbeing of PwD (Kitwood, 1997).

In our study, better expression of treatment choice was shown for individuals presenting depression. This result is in line with the literature highlighting that depressed people may have preserved decision-making capacities (Appelbaum and Grisso, 1995; for review see Hindmarch et al., 2013) and awareness of condition (Mograbi and Morris, 2014; Bertrand et al., 2016). An explanation can be found in the depressive realism theory, which argues that depression contributes to a more realistic judgment, as opposed to the normal positive or optimistic biases that are associated with an euthymic mood state (Dobson and Franche, 1989; Taylor, 1989).

Results showing that the presence of mania decreased the capacity on the *reasoning* section are consistent with findings in the literature showing executive function deficits in bipolar disorder, especially during mania (Dixon et al., 2004; Mur et al., 2007). Preserved executive functions, including abilities such as cognitive flexibility, problem-solving, planning and inhibition, appear essential in the reasoning section of the MacCAT-T, in which patients have to consider treatment alternatives and compare the consequences of these treatments. Previous studies exploring the relationship between specific neuropsychological abilities and medical decision-making showed that tests evaluating executive functioning correlated with the reasoning component of treatment consent capacity in clinical and healthy populations (for review, see Palmer and Savla, 2007). Additionally, mania has been associated with reduced awareness about the condition (de Assis da Silva et al., 2015a,b; 2016), including in patients with AD (Migliorelli et al., 1995). Lack of awareness about the condition may impact negatively reasoning about the treatment.

Some potential limitations of the current study must be considered. First, the absence of a control group may limit the interpretation of the results. Nevertheless, the lack of a control group is a feature of the current study, considering that we are exploring neuropsychiatric symptoms in dementia and their impact on decision making. Additionally, data from previous studies were reported, providing comparison points. Second, additional data from neuropsychological testing would have been useful to support some of the potential cognitive explanations proposed above. Third, because of statistical analysis limitation due to the sample size, the severity and the frequency of the neuropsychiatric symptoms were not taken into account. Fourth, we used DSM-IV-TR diagnosis criteria, which have been criticized by their insufficient diagnostic specificity. Nonetheless, DSM-IV-TR criteria have been used thoroughly in dementia research and this is a limitation that our study shares with a very large number of publications in the field of dementia studies. Finally, the present study did not consider the possible interaction of multiple neuropsychiatric symptoms in relation to decision-making capacity. Specifically in the case of apathy and depression, which have been shown to be both related to expression of a choice in the present study, although there is some overlap between the conditions, recent research has emphasized how these are separate entities (Aalten et al., 2008; Robert et al., 2009; Spalletta et al., 2010; Selbæk and Engedal, 2012). Future studies should explore the impact of multiple neuropsychiatric symptoms on the capacity to consent treatment.

Most studies in the literature focused on the cognitive predictors of decision-making capacity in dementia. However, our study stresses also a relationship between BPSD and treatment consent capacity in AD patients. These findings may have important clinical implications. Indeed, in addition to improving patients and caregivers’ quality of life, strategies aiming to reduce behavioral disturbances may lead to better decisional abilities for patient with dementia, which consequently will increase patient’s autonomy and well-being. To the best of your knowledge, this is the first study exploring directly the relationship between BPSD and consent to treatment capacity in AD patients. Therefore, future research is needed, especially to distinguish the impact of both cognitive and behavioral deficits on decision-making capacity in AD. Studies using a longitudinal design may be useful in order to understand the direction of the relationship BPSD/decision-making capacity.

## Author Contributions

MCND, DCM, JL and JL-F conceived the study design. RLS and EB were responsible for data collection. ED and DCM performed the data analysis. EB, DCM and ED drafted the manuscript. All authors revised and approved the final manuscript and are accountable for all aspects of the work.

## Conflict of Interest Statement

The authors declare that the research was conducted in the absence of any commercial or financial relationships that could be construed as a potential conflict of interest. The reviewer SB and handling Editor declared their shared affiliation, and the handling Editor states that the process nevertheless met the standards of a fair and objective review.
